# The AMerican PREGNANcy Mother–Child CohorT: description and prevalence of baseline outcomes and medication dispensing

**DOI:** 10.3389/fphar.2025.1608403

**Published:** 2025-08-05

**Authors:** Lisiane F. Leal, Odile Sheehy, Jessica Gorgui, Anick Bérard

**Affiliations:** ^1^ Research Center, CHU Sainte-Justine, Montreal, QC, Canada; ^2^ Faculty of Pharmacy, University of Montreal, Montreal, QC, Canada; ^3^ Faculty of Medicine, Université Claude Bernard Lyon, Lyon, France

**Keywords:** AMerican-PREGNANcy mother–child cohorT, pregnancy identification, medication use in pregnancy, real-world data, multi-cohort studies, administrative claims data, pharmacoepidemiology, maternal-child linkage

## Abstract

**Purpose:**

This study aims to present the AMerican PREGNANcy Mother–Child CohorT (AM-PREGNANT) and its maternal and linked-child characteristics.

**Methods:**

AM-PREGNANT was built using the Merative™ MarketScan® Commercial Database. We updated and implemented a hierarchical algorithm using ICD-9-CM and ICD-10-CM codes to identify pregnancies in individuals aged 15–45 years (2003–2021). A unique family identifier linked mothers to their children. Enrollment required continuous coverage for 90 days before, during, and 42 days after pregnancy for the mothers and 1 year after birth for the linked children. Pregnancy outcomes were categorized as deliveries, spontaneous abortions, and induced abortions. We characterized AM-PREGNANT (2004–2020) by sociodemographic factors, pregnancy history, comorbidities, and medication dispensing by pregnancy outcome. Medication dispensing, identified through filled prescriptions using drug claims, was analyzed for the 90 days before pregnancy until the last menstrual period (LMP), throughout pregnancy, and from delivery through the postpartum period. Linked children were assessed for low birth weight (LBW), preterm birth, congenital malformations, and other characteristics. Maternal and gestational age distributions were compared with United States (US) national estimates.

**Results:**

We identified 7,991,200 pregnancies from 6,079,647 persons (2003–2021). Applying continuous enrollment criteria and restricting the study period to 2004–2020 resulted in 4,767,208 pregnancies. Of these, 76.9% resulted in deliveries, 17.3% were spontaneous abortions, and 5.9% were induced abortions. The established linked mother–child cohort comprises 2,578,990 pregnancies. The mean maternal age in the linked mother–child cohort was 30.4 years (SD, 5.4). The mean gestational age at delivery was 38.6 weeks. Infections were the most prevalent maternal comorbidity (11.8%). Among deliveries, the prevalence of medication dispensing in mothers before, during, and after pregnancy were 63.2%, 88.7%, and 82.9%, respectively. Among linked children, 52.1% were male, 12.0% were preterm, and 4.5% had low birth weight. The prevalence of major congenital malformations was 13.1%. The characteristics of children with continuous enrollment were similar to those without, except for medication dispensing during the first year of life (62.9% vs. 45.6%). Both maternal and gestational age distributions of AM-PREGNANT were comparable to the US national estimates.

**Conclusion:**

AM-PREGNANT is a valuable cohort for studying medication safety in mothers and children. Strict enrollment criteria ensured reliable data, minimizing the risk of misclassification. This cohort is a key resource for multi-country perinatal pharmacoepidemiological studies.

## 1 Introduction

Over the past two decades, a steady increase in prescription medication use during pregnancy, specifically in the first trimester, has been observed (Werler et al., 2023). This rise is mainly attributed to the increasing maternal age at the first pregnancy and the higher prevalence of chronic conditions present in persons of reproductive age, such as depression and hyperglycemia (Hayes et al., 2020). Consequently, medication use during pregnancy has become inevitable in persons of childbearing age. Despite that, there is a notable lack of evidence-based information available for healthcare providers and pregnant persons or those wishing to become pregnant to assist them in their complex decision-making process, which is a growing public health concern.

Although progress has been made in advancing therapeutic research during pregnancy, pregnant individuals continue to be underrepresented in clinical trials (Yakerson, 2019; Wesley et al., 2021; Sewell et al., 2022). Electronic healthcare claims data have proven to be a feasible and reliable resource for assessing medication safety during pregnancy (Davis et al., 2024; Thurin et al., 2022; Huybrechts et al., 2019; European Network of Centres for Pharmacoepidemiology and Pharmacovigilance (ENCePP), 2023). These real-world data provide detailed information, namely, on medication exposure at various stages of pregnancy (i.e. trimesters and pre- and postnatal periods). Furthermore, with unique anonymized identifiers and the ability to link children to their mothers, these longitudinal population cohorts allow researchers to evaluate the impact of *in utero* medication exposure on both maternal and child health outcomes (Thurin et al., 2022; Huybrechts et al., 2019). These mother–child cohorts offer a unique opportunity to investigate a broad range of rare exposures and outcomes with precision due to their large sample sizes (Huybrechts et al., 2019; Su et al., 2023). Therefore, the use of secondary data becomes invaluable in studying existing and newly introduced medications for postmarketing surveillance to supplement current evidence for decision-making (Wesley et al., 2021; Huybrechts et al., 2019).

Particularly, claims and other large healthcare utilization databases have been used by several groups, and algorithms to identify pregnancies, gestational age, and linkage to infants have been developed and described before, with several studies conducted using the US claims data sources (Ailes et al., 2016; MacDonald et al., 2019; Weaver et al., 2023; Moll et al., 2021; Sumner et al., 2021; Ailes et al., 2023; Matcho et al., 2018). The Sentinel System in the US (Lyons et al., 2024) and the adoption of the Observational Medical Outcomes Partnership (OMOP) Common Data Model (CDM) across Europe to create a federated network (Thurin et al., 2022) have improved over time. Similar advances have been made in Canada (Bérard and Sheehy, 2014; Bérard et al., 2022). These initiatives have improved the quality of observational research in pregnancy, thus increasing the validity of study findings and reducing the knowledge gap for making informed decisions regarding medication use during pregnancy and outcomes in exposed children.

The Canadian Mother–Child Cohort (CAMCCO) initiative on drug Safety in pregnancy), based on the Quebec Pregnancy Cohort (QPC) model, was established to support the creation of harmonized provincial mother–child cohorts across Canada (Bérard and Sheehy, 2014; Bérard et al., 2022). However, US and Canadian insurance claims data differ mainly in the scope and structure, with US data being fragmented across private insurers and Canadian data being more centralized and population-based due to its publicly funded healthcare system. Leveraging Canadian and US claims data represents a great opportunity to address research questions on the safety and effectiveness of medication use during pregnancy. It is important to note that when referring to the US claims data, since the same data sources change providers and health and insurance policies change over time, definitions must be updated and codes have to be adapted in order to create new cohorts to be used either individually or as part of multi-country studies. Furthermore, demonstrating the reproducibility of previous work across different settings is crucial to ensure the reliability of cohorts used in individual or pooled analyses.

In this regard, our objective is to create the AMerican PREGNANcy Mother–Child CohorT (AM-PREGNANT) using the Merative™ MarketScan® research databases (hereafter referred to as MarketScan®). We adapted previously described algorithms and carefully updated ICD-9 and ICD-10 definitions. To demonstrate that cohort creation is feasible, characteristics are reliable, and the cohort can be used in multi-country studies, we present the baseline characteristics of pregnant persons and their children included in AM-PREGNANT, in addition to the prevalence of medication dispensing in pregnant individuals and their linked children. A descriptive comparison of summary statistics is also presented, referencing US estimates of maternal and gestational age distributions.

## 2 Materials and methods

### 2.1 Data source

To create AM-PREGNANT, we used the MarketScan® Commercial Database from Merative® L.P. (formerly IBM Watson Health), which includes de-identified, patient-specific health data of employees/workers, their spouses, and dependents who are covered by employer-sponsored private health insurance in the US. This database contains data on over 203 million individuals covered annually by medium- and large-sized employers and health plans. MarketScan® provides a nationally representative sample of patients with employer-provided health insurance in the US, making it an appropriate data source for perinatal pharmacoepidemiological studies.

### 2.2 Cohort development

MarketScan® is a claims database in which the date of onset of pregnancy is not explicitly recorded. AM-PREGNANT was built upon previous literature that described frameworks, diagnoses, and procedure code definitions used to assemble linked mother–child pregnancy cohorts (Ailes et al., 2016; MacDonald et al., 2019; Moll et al., 2021; Sumner et al., 2021; Ailes et al., 2023; Matcho et al., 2018; Sa et al., 2020; Hornbrook et al., 2007). We conducted a literature review on pregnancy cohort creation using similar data sources and anchored our approach in the six-step algorithm published by MacDonald et al. (2019).

Moreover, the following improvements were implemented while creating AM-PREGNANT: (i) review and translation of ICD-9 codes into ICD-10 codes using AHRQ MapIT Software (Agency for Healthcare Research and Quality (AHRQ), 2023a); (ii) incorporation of the reviewed definitions into the six-step algorithm; (iii) inclusion of ICD-10 codes for defining gestational age; and (iv) comparison of distributions, by region, of average maternal age and gestational age with the US national estimates from the Centers for Disease Control and Prevention (CDC) Wide-ranging Online Data for Epidemiologic Research (WONDER) (Centers for Disease Control and Prevention (CDC), 2023a; Centers for Disease Control and Prevention (CDC), 2023c). The list of codes, definitions, approach, and rationale for SAS programming is available in Supplementary File S1.

One important adjustment over the MacDonald et al. (2019) approach was to incorporate and prioritize ICD-10 gestational age based on specific prenatal codes from the literature review (denoting the exact weeks of gestation, e.g., ICD-10 CM Z3A.08 to Z3A.42 codes). Furthermore, when codes for gestational age were absent, we assigned 39 weeks for live births, 28 weeks for stillbirths, 35 weeks for mixed births, 8 weeks for spontaneous abortions, 10 weeks for induced/planned abortions, and 9 weeks for unspecified abortions, following the approach of MacDonald et al. (2019). The first day of the last menstrual period (LMP) was estimated by subtracting the gestational age from the date of pregnancy outcome. The assignment of the above-mentioned gestational ages has been previously validated and demonstrated to provide optimal estimates of the LMP when gestational age was not recorded (Hornbrook et al., 2007; Margulis et al., 2013; Margulis et al., 2023). The complete list of specific prenatal codes used to define the gestational age is provided in Supplementary File S1. To compare our adapted approach with previous validated definitions, we demonstrated the gestational age distribution compared to the CDC WONDER estimates (detailed further) (Centers for Disease Control and Prevention (CDC), 2023c).

To define continuous enrollment for mothers, we used the 2003–2021 enrollment detail files to calculate a person’s enrollment duration within the 90 days before LMP to 42 days after the end of the pregnancy. The use of 42 days after the end of the pregnancy refers to the postpartum period, according to the (World Health Organization (WHO), 2010). The first 24–48 h are the most critical for the mother and the baby, with the risks of ill health and death being high within the postpartum/postnatal period (World Health Organization (WHO), 2010). The decision was made to capture accurate information for the dyad when using this cohort. We allowed for a single 30-day gap in enrollment during that time period, and all persons who did not meet this criterion were considered not continuously enrolled. No continuously enrolled individuals were subsequently excluded from our analyses. Among the continuously enrolled persons with pregnancies ending in live births, mixed births, or stillbirths, where linkage with at least one child was possible, we further required that newborns also be continuously enrolled in their medication and health insurance plan for a minimum of 365 days after birth. Similarly, children were allowed a single 30-day gap in enrollment, which was ascertained using the enrollment detail files. The requirement of continuous enrollment was applied to ensure data completeness (defined as the presence of a claim or prescription fill during the follow-up) for individuals included in AM-PREGNANT. This strategy has previously been used in pregnancy cohorts using MarketScan® data (MacDonald et al., 2019; Ailes et al., 2023) to ensure complete follow-up and complete ascertainment of medication dispensing, diagnoses, and procedures for mothers, while also ascertaining the major congenital birth defects in children (Huybrechts et al., 2019).

### 2.3 Baseline characteristics, comorbidities, and medication dispensing

Pregnancies that ended between 1 January 2004 and 31 December 2020 were retained to describe their baseline characteristics. This decision was made to allow all pregnancies the possibility of reaching full-term within the study period and allow at least 365 days of follow-up for the children born by December 2020, according to the time-frame of data availability (2003–2021).

The calendar year of the pregnancy, region of residence, gestational age, and maternal age (continuous and categorized into <20, 20–34, 35–40, and >40 years) were measured at the end of pregnancy. All other characteristics, including previous multi-fetal pregnancies, previous cesarean delivery, alcohol/substance abuse, and comorbidities, were measured in the 90 days before LMP until the end of pregnancy. In MarketScan®, the region of residence is defined as the geographic region of the employee’s residence and is categorized into five groups: northeast, north central, south, west, and unknown. The studied maternal comorbidities were thyroid disorders, depression, hypertension, asthma, diabetes, epilepsy, autoimmune diseases, infections, obesity, and renal diseases, identified using the International Classification of Diseases, Ninth and Tenth Revision (ICD-9 and ICD-10) codes. We adopted harmonized definitions of covariates and outcomes with Canadian cohorts for both pregnant persons and children (Supplementary File S2). To date, other comorbidities could have been defined using MarketScan® data (Merative, 2025). However, for this study, we focus on common risk factors that have been examined in previous multi-country studies and harmonized across pregnancy-linked cohorts (Bérard and Sheehy, 2014; Bérard et al., 2022).

Maternal medication dispensing was defined as having at least one filled prescription (identified via National Drug Codes [NDCs]) during a specified time window or a prescription filled prior to the window with a duration that overlapped it. The assessment windows were as follows: (i) the 90 days prior to the start of pregnancy (LMP–90 days); (ii) during pregnancy (from the LMP to the pregnancy end date); and (iii) postpartum (the 42-day period following the pregnancy end date). The pregnancy period was further divided into trimesters: first trimester (LMP to 98 gestational days), second trimester (99 to 182 gestational days), and third trimester (183 gestational days to the end of pregnancy), regardless of pregnancy outcome. We defined gestational use of medications as having at least one filled prescription (identified through National Drug Codes—NDCs) during the specified time-windows or a prescription filled before the time-window with a duration that overlapped the window. The prevalence of medication dispensing was estimated as the proportion of pregnancies with any filled prescription medication among all pregnancies. The prevalence of medication dispensing with and without vitamins and combinations among deliveries was assessed by the time-window of exposure. Prevalence was also estimated by medication class. Among the 28 medication classes (mapped from the ranges of Red Book® Therapeutic Class Codes; Supplementary File S2), we presented the prevalence of medication dispensing for the top 10 most frequently filled medication classes.

For children, medication dispensing was estimated as the proportion of any prescription medication filled within the first year of life. The prevalence of medication dispensing was also presented for the top 10 most commonly used medication classes.

### 2.4 Pregnancy and children’s outcomes

Pregnancy outcomes were assessed at the end of pregnancy. For harmonization purposes, we aimed for comparison with Canadian cohorts (Bérard and Sheehy, 2014; Bérard et al., 2022). Pregnancy outcomes were categorized into (Werler et al., 2023) deliveries, which include live births, stillbirths, and mixed births (Hayes et al., 2020); spontaneous abortions; and (Yakerson, 2019) induced/planned abortions, which include induced/planned abortions and unspecified abortions.

Preterm birth was measured in the linked-child cohort. We also measured preterm birth using pregnancies ending in a live birth to compare with Canadian estimates. Preterm birth was based upon the definition employed by WHO, which defines it as a birth occurring before 37 weeks of gestation, measured from the first day of the LMP (WHO, 2023). We also examined sub-categories of preterm births based on gestational age: (i) extremely preterm (less than 28 weeks of gestation); (ii) very preterm (28 to less than 32 weeks of gestation); and (iii) moderate to late preterm (32–37 weeks of gestation) (WHO, 2023). The prevalence of preterm births was calculated as a proportion of (i) all live births and (ii) all linked children, with results further stratified by child enrollment status.

Low birth weight (LBW) and major congenital malformations were also measured among linked children. We identified LBW and major congenital malformations within inpatient and outpatient files of linked children who were continuously enrolled for at least 365 days after the end of pregnancy. LBW is defined by the WHO as a birth weight of less than 2,500 g (up to and including 2,499 g) (World Health Organization (WHO), 2023a). As weight at birth is not recorded in the MarketScan® database, we defined LBW as at least one ICD-9 or ICD-10 code for LBW according to the Agency for Healthcare Research and Quality (AHRQ) definition (Supplementary File S2) (Agency for Healthcare Research and Quality (AHRQ), 2023b).

Major congenital malformations were grouped into 12 organ systems: (a) circulatory; (b) musculoskeletal; (c) urinary; (d) nervous; (e) digestive; (f) integumentary; (g) respiratory systems; (h) genital organs; (i) eye, ear, face, and neck; (j) chromosomal abnormalities; (k) cleft palate and/or lip; and (l) other. We demonstrate the prevalence of major congenital malformations obtained by two definitions. Definition A required at least one ICD-9 or ICD-10 code in the first 12 months of life according to the EUROCAT classification (European Surveillance of Congenital Anomalies (EUROCAT), 2013) and previous Canadian definitions (Bérard and Sheehy, 2014; Bérard et al., 2022), while definition B required at least two ICD-9 or ICD-10 codes in different dates in the first 12 months of life. Diagnosis codes are provided in Supplementary File S2. Major congenital malformations were assessed in singleton live births using children’s inpatient and outpatient files. LBW and other characteristics, including neonatal diseases in the first 2 months of life, comorbidities in the child’s early life, and medication dispensing, were studied using the inpatient and outpatient files of both mothers and children to ensure a comprehensive record of healthcare encounters.

### 2.5 Statistical methods

We conducted descriptive analyses to summarize the cohort’s characteristics. Proportions were reported for categorical variables, while means and standard deviations (SDs) were used for continuous variables. To assess potential differences between subgroups, estimates were contrasted; however, inferential statistical tests were not performed due to the descriptive nature of the analysis (Lash et al., 2021; Lesko et al., 2022). Where appropriate, cohort characteristics and outcome measures were compared with the national estimates extracted from CDC WONDER (Centers for Disease Control and Prevention (CDC), 2023a; Centers for Disease Control and Prevention (CDC), 2023c) to provide context and highlight consistencies or discrepancies.

Characteristics and filled prescription medications were presented for the pregnancy cohort by pregnancy outcome and for the linked mother–child cohort and the non-linked cohort. For linked children, characteristics were presented overall and according to the enrollment status (i.e., continuously and non-continuously enrolled). The prevalence of preterm birth and LBW was examined by the calendar year at the end of pregnancy. Major congenital malformations were assessed within 1 year after birth. Prevalence per 100 pregnancies for the top 10 most frequently filled prescribed medication classes was reported by time windows during pregnancy (i.e. the first, second, and third trimesters). LBW and major congenital malformations were presented as absolute numbers and prevalence per 100 live births.

In addition to building on previously described algorithms (MacDonald et al., 2019; Ailes et al., 2023), we assessed the validity of our method in terms of whether the characteristics of AM-PREGNANT were consistent with (or differed from) the US population estimates. The assessment of external validity using proper methods has been recently published (Webster-Clark et al., 2023; Webster-Clark et al., 2024a). For this study, we performed a descriptive comparison of summary statistics using the US national estimates, a previously described approach (Taylor et al., 2022), to assess the validity of AM-PREGNANT characteristics. To compare the trends of the characteristics of our cohort with the US estimates, we obtained estimates from the CDC WONDER (Centers for Disease Control and Prevention (CDC), 2023a). CDC WONDER collects data on several indicators, including births and fetal deaths. For the current study, we selected the natality information by census region of residence and year for the following measures: the average age of mothers and the average LMP gestational age, which were measured for live births only. We used the estimates stratified by regions for comparisons.

To assess the similarities between AM-PREGNANT and previously established Canadian mother–child cohorts, we compared the proportions of the main outcomes and medication dispensing among the previously published cohorts (Bérard and Sheehy, 2014; Bérard et al., 2022). Definitions of prematurity (and its subcategories), LBW, multiplicity, major congenital malformations, and medication dispensing during pregnancy from the Canadian cohorts were adopted to describe AM-PREGNANT.

Access to and analysis of MarketScan® were performed from November 2022 to November 2023. All analyses were conducted using SAS v9.4 (SAS Institute Inc., North Carolina, United States).

### 2.6 Ethics statement

This study was approved by the CHU Sainte-Justine’s Ethics Committee. Only anonymized data were available and analyzed.

## 3 Results

**FIGURE 1:**
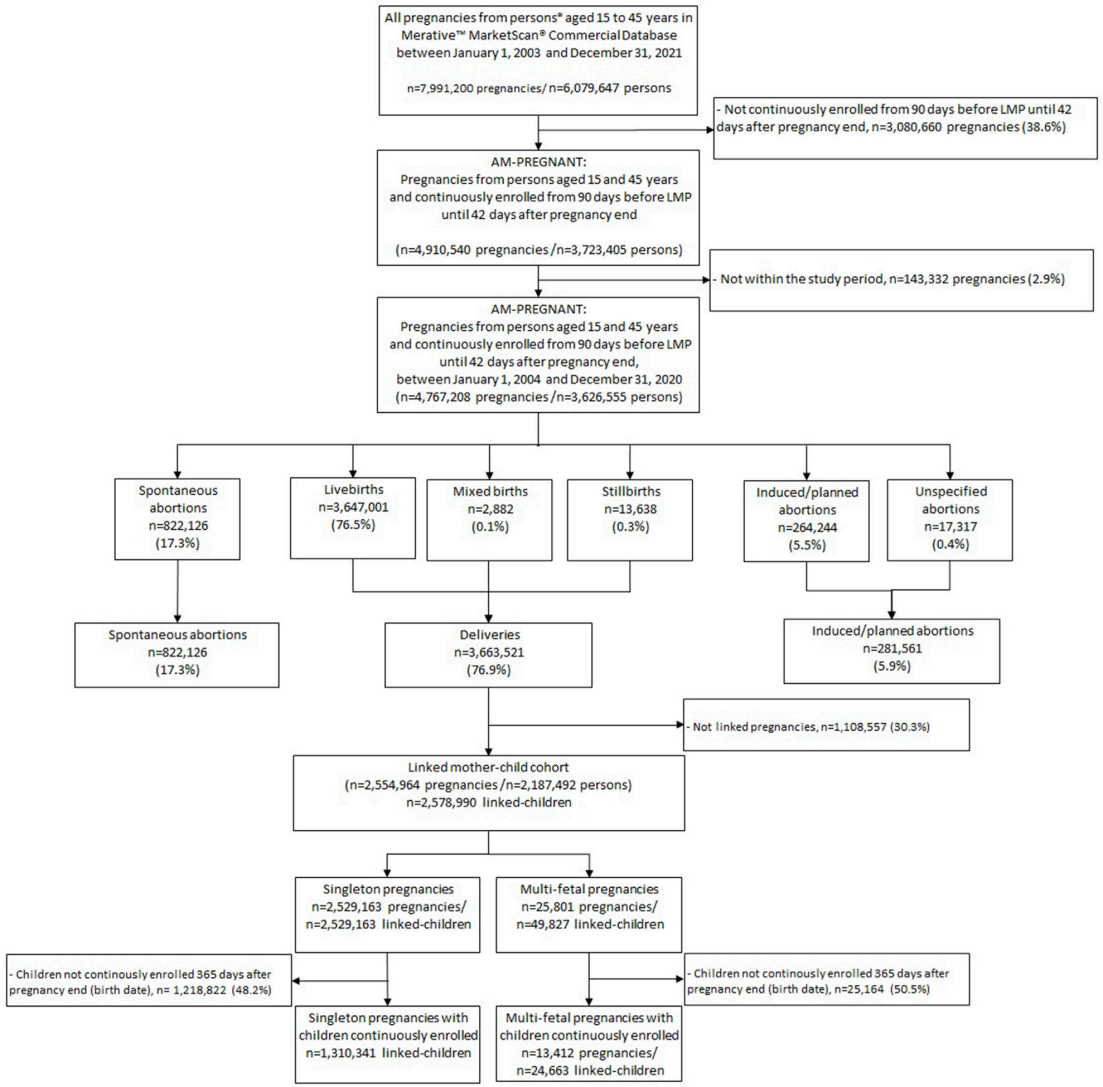
Selection of pregnancies included in AM-PREGNANT. Notes (Werler et al., 2023): mothers were required to be continuously enrolled from 90 days before the LMP to 42 days after the end of pregnancy, and children’s enrollment linked to these pregnancies was of at least 365 days after the end of pregnancy (date of birth) (Hayes et al., 2020). The study period for AM-PREGNANT description was defined between 1 January 2004 and 31 December 2020 to allow the pregnancies to reach full-term within the study period and for allowing at least 365 days of follow-up for children born by December 2020.

We identified 7,991,200 pregnancies from 6,079,647 persons aged 15–45 years between 1 January 2003 and 31 December 2021. The selection process of pregnancies included in AM-PREGNANT is shown in Figure 1. AM-PREGNANT included 4,767,208 pregnancies among 3,626,555 continuously enrolled persons, and the study period was between 1 January 2004 and 31 December 2020 (Figure 1). Deliveries represented 76.9% of pregnancy outcomes, followed by 17.3% of spontaneous abortions and 5.9% of induced/planned abortions (Figure 1).

The linked mother–child cohort was composed of 2,554,964 pregnancies linked to 2,578,990 children. Among them, 1,310,341 were continuously enrolled children linked to singleton pregnancies, while 24,663 children were linked to 13,412 multi-fetal pregnancies (Figure 1).

### 3.1 Pregnancy and children characteristics

The number and proportion of pregnancies overall and stratified by pregnancy outcomes varied over time, with a slight increase in the percentage of pregnancies observed between 2007 and 2012, followed by a return to the percentage observed at the beginning of the study period. However, the proportion of induced/planned abortions decreased by half after 2014 (Table 1). The majority of pregnancies included in AM-PREGNANT were in the southern region (40.3%), followed by the north central (22.0%), west (19.2%), and northeast (17.0%) regions (Table 1). The distribution followed the same pattern when stratified by pregnancy outcome, with the exception of induced/planned abortions, in which the greatest proportion was identified in the northeast region (36.7%) (Table 1). The overall average maternal age was 30.6 years (SD: 5.7), while an older maternal age among pregnancies with spontaneous abortions was observed (31.9 years, SD 6.3), along with a younger maternal age among those with induced/planned abortions (29.2 years, SD 7.2) (Table 1). The overall average gestational age within AM-PREGNANT was 31.7 weeks (SD 12.6), with 38.6 weeks (SD 2.0) for deliveries (Table 1). Overall, diabetes, depression, and hypertensive disorders were the top three most frequent comorbidities affecting 12.9%, 10.9%, and 9.9% of pregnancies, respectively. Pregnancies ending in induced/planned abortions had the highest proportion of depression among all pregnancies (Table 1). Alcohol and other substance abuse was recorded in 1.4% of all pregnancies, with a similar distribution among the pregnancy outcomes, although we acknowledge the high potential for underreporting, as this variable is defined using diagnostic codes (Table 1).

**TABLE 1:** Maternal characteristics of AM-PREGNANT (from the Merative^™^ MarketScan^®^ Commercial Database, US) by pregnancy outcome^a^ (2004–2020).

Characteristic	All pregnancies 2004–2020^b^	Deliveries	Spontaneous abortions	Induced/planned abortions
	(n = 4,767,208)	(n = 3,663,521)	(n = 822,126)	(n = 281,561)
Calendar year at the end of pregnancy				
2004	139,529 (2.9%)	100,323 (2.7%)	21,142 (2.6%)	18,064 (6.4%)
2005	181,309 (3.8%)	134,174 (3.7%)	25,081 (3.1%)	22,054 (7.8%)
2006	180,333 (3.8%)	126,022 (3.4%)	40,642 (4.9%)	13,669 (4.9%)
2007	266,222 (5.6%)	200,951 (5.5%)	49,131 (6.0%)	16,140 (5.7%)
2008	301,949 (6.3%)	217,605 (5.9%)	60,724 (7.4%)	23,620 (8.4%)
2009	392,236 (8.2%)	294,716 (8.0%)	70,827 (8.6%)	26,693 (9.5%)
2010	379,978 (8.0%)	287,795 (7.9%)	67,331 (8.2%)	24,852 (8.8%)
2011	414,907 (8.7%)	315,108 (8.6%)	72,982 (8.9%)	26,817 (9.5%)
2012	452,096 (9.5%)	348,905 (9.5%)	76,145 (9.3%)	27,046 (9.6%)
2013	364,270 (7.6%)	281,845 (7.7%)	59,408 (7.2%)	23,017 (8.2%)
2014	365,363 (7.7%)	282,922 (7.7%)	59,650 (7.3%)	22,791 (8.1%)
2015	260,957 (5.5%)	206,453 (5.6%)	41,664 (5.1%)	12,840 (4.6%)
2016	273,101 (5.7%)	224,290 (6.1%)	42,562 (5.2%)	6,249 (2.2%)
2017	240,617 (5.1%)	196,255 (5.4%)	38,739 (4.7%)	5,623 (2.0%)
2018	222,146 (4.7%)	179,209 (4.9%)	37,794 (4.6%)	5,143 (1.8%)
2019	175,156 (3.7%)	142,251 (3.9%)	29,236 (3.6%)	3,669 (1.3%)
2020	157,039 (3.3%)	124,697 (3.4%)	29,068 (3.5%)	3,274 (1.2%)
Region				
Northeast	809,990 (17.0%)	563,995 (15.4%)	142,682 (17.4%)	103,313 (36.7%)
North Central	1,049,807 (22.0%)	832,150 (22.7%)	176,636 (21.5%)	41,021 (14.6%)
South	1,923,236 (40.3%)	1,518,741 (41.5%)	336,929 (41.0%)	67,566 (24.0%)
West	916,461 (19.2%)	697,387 (19.0%)	153,245 (18.6%)	65,829 (23.4%)
Unknown	67,714 (1.4%)	51,248 (1.4%)	12,634 (1.5%)	3,832 (1.4%)
Maternal age at the end of pregnancy, years				
Mean (SD)	30.6 (5.7)	30.4 (5.4)	31.9 (6.3)	29.2 (7.2)
<20	164,927 (3.5%)	109,525 (3.0%)	27,745 (3.4%)	27,657 (9.8%)
20–34	3,402,679 (71.4%)	2,723,680 (74.4%)	499,190 (60.7%)	179,809 (63.9%)
35–40	1,021,976 (21.4%)	739,084 (20.2%)	224,814 (27.4%)	58,078 (20.6%)
>40	177,626 (3.7%)	91,232 (2.5%)	70,377 (8.6%)	16,017 (5.7%)
Estimated gestational age, weeks				
Mean (SD)	31.7 (12.6)	38.6 (2.0)	8.6 (1.4)	10.2 (2.0)
Multi-fetal pregnancies	104,698 (2.2%)	96,664 (2.6%)	6,434 (0.8%)	1,600 (0.6%)
Previous cesarean delivery	175,941 (3.7%)	169,505 (4.6%)	5,039 (0.6%)	1,397 (0.5%)
Alcohol/substance abuse	64,662 (1.4%)	54,757 (1.5%)	5,537 (0.7%)	4,368 (1.6%)
Tobacco use	45,890 (1.0%)	37,678 (1.0%)	5,715 (0.7%)	2,497 (0.9%)
Maternal comorbidity				
Thyroid disorders	400,606 (8.4%)	332,924 (9.1%)	55,952 (6.8%)	11,730 (4.2%)
Depression	517,159 (10.9%)	397,742 (10.9%)	86,903 (10.6%)	32,514 (11.6%)
Hypertension	471,823 (9.9%)	421,474 (11.5%)	38,968 (4.7%)	11,381 (4.0%)
Asthma	376,682 (7.9%)	312,958 (8.5%)	47,832 (5.8%)	15,892 (5.6%)
Diabetes	615,548 (12.9%)	574,126 (15.7%)	34,727 (4.2%)	6,695 (2.4%)
Epilepsy	13,927 (0.3%)	11,454 (0.3%)	1,772 (0.2%)	701 (0.3%)
Auto-immune diseases	85,692 (1.8%)	68,260 (1.9%)	13,437 (1.6%)	3,995 (1.4%)
Infections	476,735 (10.0%)	431,539 (11.8%)	30,229 (3.7%)	14,967 (5.3%)
Obesity	239,271 (5.0%)	219,816 (6.0%)	15,112 (1.8%)	4,343 (1.5%)
Renal diseases	31,920 (0.7%)	30,067 (0.8%)	1,339 (0.2%)	514 (0.2%)
Medication dispensing (overall)	3,673,665 (77.1%)	2,900,679 (79.2%)	582,661 (79.9%)	190,325 (67.6%)
90 days before pregnancy	2,395,989 (65.2%)	1,834,093 (63.2%)	430,152 (73.8%)	131,744 (69.2%)
During pregnancy	3,183,727 (86.7%)	2,573,443 (88.7%)	456,890 (78.4%)	153,394 (80.6%)
First trimester	2,722,186 (85.5%)	2,113,450 (82.1%)	456,152 (99.8%)	152,584 (99.5%)
Second trimester	1,965,935 (61.7%)	1,876,847 (72.9%)	60,504 (13.2%)	28,584 (18.6%)
Third trimester	1,920,988 (60.3%)	1,919,418 (74.6%)	533 (0.1%)	1,037 (0.7%)
42 days after pregnancy	3,041,109 (82.8%)	2,405,181 (82.9%)	479,438 (82.3%)	156,490 (82.2%)

Abbreviation: SD, standard deviation

^a^ Pregnancy outcomes are as follows: livebirth, stillbirth, and mixed births grouped into delivery, induced/planned abortions, and unspecified abortion grouped into induced/planned abortions and spontaneous abortions.

^b^ May represent more than one pregnancy per person.

Overall, 77.1% of pregnancies had at least one prescription medication filled (including vitamins and topical medications) from 90 days before LMP until 42 days after the end of pregnancy. The assessment of filled prescription medication by pregnancy time-windows demonstrated that 65.2% of pregnancies had a prescription filled in the 90 days before the LMP, 86.7% during pregnancy, and 82.8% in the 42 day-period after the end of the pregnancy. Among those with at least one prescription medication filled during pregnancy, the first trimester was the period with the highest prevalence (85.5%), with lower estimates observed in the second trimester (61.7%) and third trimester (60.3%) (Table 1). For pregnancies ending in delivery, the exclusion of vitamins and their combinations did not drastically change the estimates of prescription dispensing during pregnancy (88.7% vs. 84.1%, Supplementary File S3; Table 2). For pregnancies ending in spontaneous abortions and induced/planned abortions, prevalence rates of prescription medications filled during the first trimester were 99.8% and 99.5%, respectively (Table 1). For spontaneous and planned abortions, the prevalence rates of prescription medication filled during the second and third trimesters were 13% and 19%, respectively. Medication dispensing observed in the third trimester for the non-live birth outcomes represents prescriptions that overlap with the beginning of the time window.

**TABLE 2:** Linked-children characteristics in AM-PREGNANT (from the Merative^™^ MarketScan^®^ Commercial Database, US) by enrollment status (2004–2020).

Characteristic	Children overall	Children continuously enrolled	Children not continuously enrolled
Pregnancies, n	4,767,208	1,323,753	1,232,317
Linked children, n	2,578,990	1,335,004	1,243,986
Children’s characteristics at birth			
Male sex	1,040,667 (52.4%)	620,863 (52.1%)	419,804 (52.9%)
Preterm (less the 37 gestational weeks completed)	307,700 (11.9%)	159,673 (12.0%)	148,027 (11.9%)
Extremely preterm (<28 weeks)	5,452 (2.2%)	2,059 (1.6%)	3,393 (2.8%)
Very preterm (28-<32 weeks)	16,822 (6.8%)	8,132 (6.5%)	8,690 (7.2%)
Moderate to late preterm (32-<37 weeks)	223,608 (90.9%)	115,545 (91.9%)	108,063 (89.9%)
Low birth weight (less than 2,500 g at birth)	121,837 (4.7%)	60,249 (4.5%)	61,588 (5.0%)
Neonatal diseases in the two first months of life			
Hypoglycemia	57,329 (2.2%)	24,343 (1.8%)	32,986 (2.7%)
Chronic kidney disease	2,033 (0.1%)	923 (0.1%)	1,110 (0.1%)
Liver disease	1,601 (0.1%)	1,903 (0.1%)	698 (0.1%)
Immunodeficiency disease	739 (0.03%)	347 (0.03%)	392 (0.03%)
Comorbidities in child early life (6 months of life)			
Fetal alcohol syndrome	120 (0.00%)	44 (0.00%)	76 (0.01%)
Meningitis	3,873 (0.2%)	2,018 (0.2%)	1,855 (0.2%)
Whooping cough	1,960 (0.1%)	1,175 (0.1%)	785 (0.1%)
Measles	35 (0.00%)	19 (0.00%)	16 (0.00%)
Seizure disorders	4,029 (0.2%)	2,328 (0.2%)	1,701 (0.1%)
Hearing loss	49,486 (1.9%)	27,652 (2.1%)	21,834 (1.8%)
Middle ear infections (otitis media)	299,987 (11.6%)	194,613 (14.6%)	105,374 (8.5%)
Medication dispensing in the first year of life	1,406,313 (54.5%)	839,035 (62.9%)	567,278 (45.6%)

**FIGURE 2:**
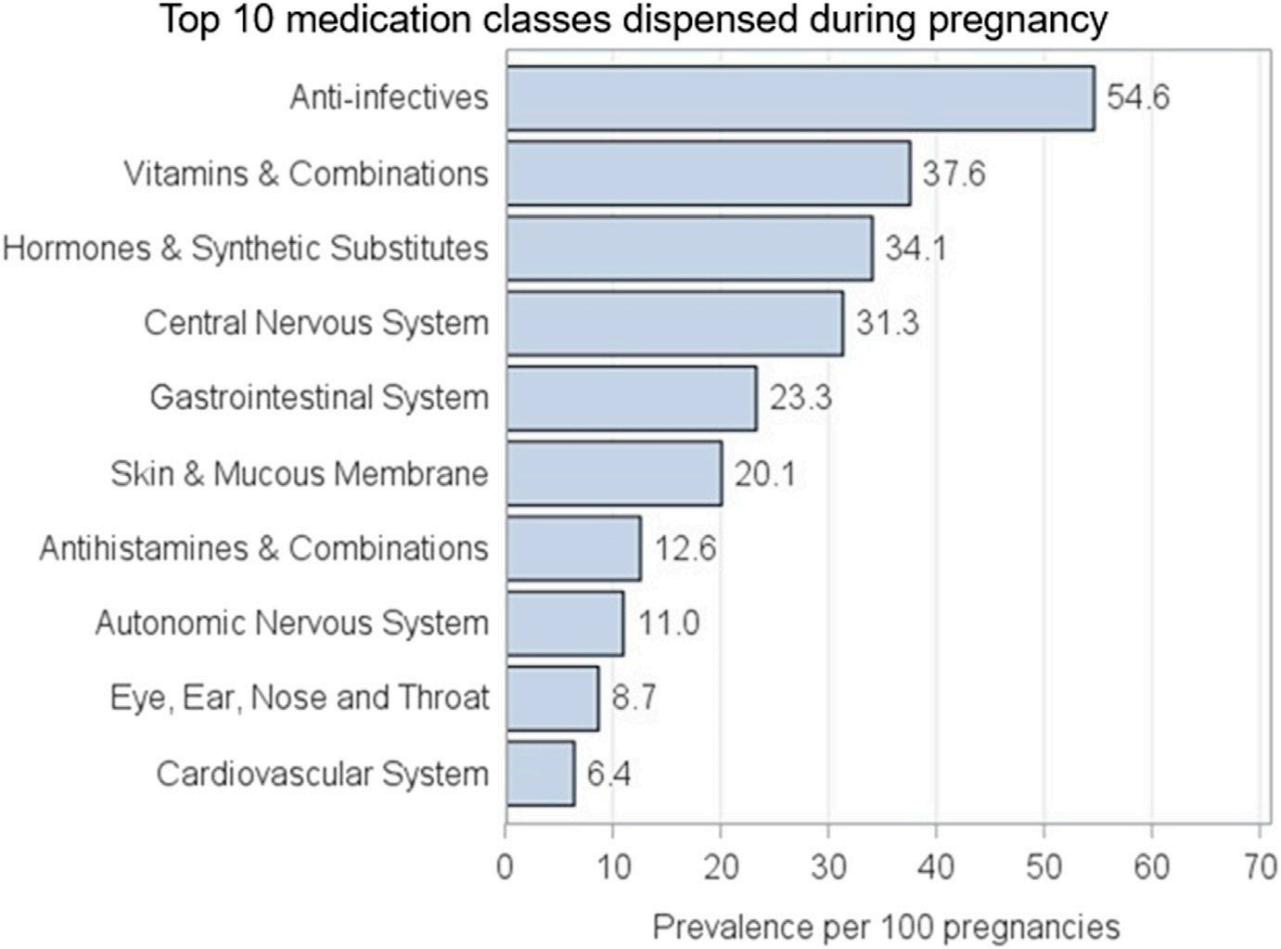
AM-PREGNANT top 10 classes of medication most commonly used during pregnancy.

The top 10 most frequently filled prescription medication classes during pregnancy are shown in Figure 2. Overall, anti-infectives were the leading class of filled prescription medications, with more than half of pregnancies filling at least one treatment (54.6%), followed by vitamins and combinations (37.6%) and hormones and synthetic substitutes (34.1%).

Supplementary File S3 and Table 1 show the characteristics of pregnancies that were linked and non-linked to infants. The distribution of pregnancies over time and regions remained similar when comparing both groups. However, for some characteristics, accentuated differences in proportions could be observed. Non-linked vs. linked-pregnancies presented a greater proportion of mothers who are younger than 20 years old (9.0% vs. 0.4%), with higher alcohol/substance abuse (2.4% vs. 1.1%), and a greater proportion of depression (6.5% vs. 5.2%) and asthma (5.7% vs. 4.8%). On the other hand, medication dispensing assessment in all time-windows of pregnancy presented lower proportions in non-linked pregnancies (Supplementary File S3; Table 1).

Characteristics of linked children are shown in Table 2. Among all linked children, 52.4% were of the male sex and 11.9% had a preterm birth, with the majority of preterm births being moderate-to-late preterm (32–37 weeks) (90.9%) (Table 2). Low birth weight prevalence was 4.7%, and 11.6% children presented with middle-ear infection. Overall, 54.5% of linked children had at least one prescription filled within the first year of life. When comparing continuously enrolled children with those not continuously enrolled, the characteristics were similar, except for the prevalence of middle-ear infections and medication dispensing during the first year of life. Both prevalence rates were lower among those without continuous enrollment: 8.5% vs. 14.6% for otitis and 45.6% vs. 62.9% for prescription medication filled. For major congenital malformations, overall, 13.1 vs. 4.0 major congenital malformations per 100 live births were identified from definitions A and B, respectively (Table 3). For definition B, when at least two diagnosis codes were required, the most prevalent malformations by organ systems were related to the circulatory system (42.4%), followed by the musculoskeletal system (24.5%) and genital organs (15.0%) (Table 3).

**TABLE 3:** Prevalence of major congenital malformations per 100 singleton live births (n = 1,310,341)^a^.

Criterion	Definition A	Definition B
Definition summary	At least one ICD-9 or ICD-10 code in the first 12 months of life	At least two ICD-9 or ICD-10 codes in different dates in the first 12 months of life
Population^a^	Live births	Live births
Sample size (n)	1,310,341	1,310,341
Prevalence n (%)		
Any major congenital malformation	171,539 (13.1)	52,898 (4.0)
Circulatory system	60,041 (35.0)	22,420 (42.4)
Musculoskeletal system	51,409 (30.0)	12,939 (24.5)
Genital organs	33,111 (19.3)	7,953 (15.0)
Urinary system	15,976 (9.3)	5,163 (9.8)
Nervous system	13,355 (7.8)	3,056 (5.8)
Eye, ear, face and neck	6,621 (3.9)	1,360 (2.6)
Chromosomal abnormalities	5,033 (2.9)	1,896 (3.6)
Digestive system	4,147 (2.4)	375 (0.7)
Respiratory system	3,389 (2.0)	577 (1.1)
Cleft palate and/or lip	2,728 (1.6)	1,887 (3.6)
Integumentary system	2,252 (1.3)	77 (0.2)
Other	9,671 (5.6)	2,075 (3.9)

^a^ Measured among singleton linked-children with continuous enrollment.

**FIGURE 3:**
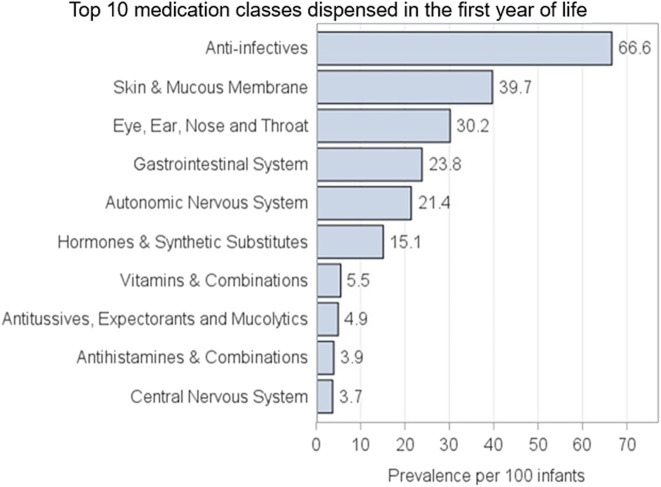
AM-PREGNANT top 10 classes of medication most commonly dispensed to linked-children in the first year of life.

Figure 3 shows the top 10 classes of prescription medication filled by children, with 66.6% of all linked children exposed to anti-infectives, followed by 39.7% exposed to skin and mucous membranes products and 30.2% to eye, ear, nose, and throat medications.

Descriptive comparisons with the US National and Canadian estimates are presented in Supplementary File S3. Figure 1 shows the distribution of the average maternal age by region for deliveries (comprising 76.5% of live births) of 30.6 years in AM-PREGNANT, which is comparable to the reported mean maternal age of 29.4 years in the US population when using the CDC WONDER database (Centers for Disease Control and Prevention (CDC), 2023c). The estimated average gestational age for deliveries (38.6 weeks) was similarly comparable to the 38.6 weeks reported for the overall US population during a similar period (Supplementary File S3; Figure 2). Comparison of outcomes among the Canadian cohorts and AM-PREGNANT revealed differences in the distribution of all compared characteristics (Supplementary File S3; Table 2).

## 4 Discussion

AM-PREGNANT represents a large and up-to-date mother–child cohort using the MarketScan® database built for contributing to perinatal pharmacoepidemiological research, including its use in multi-country studies. We demonstrated the feasibility of addressing research questions for both pregnant individuals and their children using a large sample size, with pregnancy outcomes, linked children outcomes, and medication dispensing data described over 17 years (2004–2020). Linked and non-linked pregnancies presented slight differences, which should be taken into account when defining research questions, applying methods, and interpreting the results in terms of external validity and its related concepts (i.e. target populations, generalizability, and transportability) (Webster-Clark et al., 2023). The same rationale is also needed when the study population is composed of linked children with and without continuous enrollment.

In AM-PREGNANT, maternal age at delivery was higher among linked pregnancies (32 years) than among non-linked pregnancies (28 years). This pattern aligns with the findings of Weaver et al. (2023), who reported the same age distribution for linked versus non-linked pregnancies. Similarly, Ailes et al. (2023) observed that non-linked pregnancies were more common among younger individuals (15–24 years at delivery), further supporting the association between younger maternal age and non-linkage. In this work, we also demonstrated that these pregnancies involved individuals with higher rates of alcohol and substance use, slightly poorer health—including a higher proportion of infections and depression—a lower prevalence of filled prescription medications, both overall and across time-windows of use. This information is valuable and can be used in future studies to address selection bias that could arise from different mechanisms of selection related to both the exposure and the outcome (Fox et al., 2021). Selection bias due to loss of follow-up is another limitation that could be addressed based on our cohort description. We defined a strict continuous enrollment period, allowing a maximum 30-day gap between enrollment periods for both the mother and the linked children. Ailes et al. (2023), in order to make the criterion less strict, showed that a 60-day gap would not have any impact on linked and non-linked pregnancies. We decided to maintain strict enrollment criteria for both mothers and children in order to minimize the risk of introducing selection bias. In AM-PREGNANT, in the linked-child cohort, children with a gap of enrollment greater than 30 days presented a lower prevalence of medication dispensing (45.6% vs. 62.9%). Therefore, increasing the sample size may also increase the risk of introducing both differential selection and measurement errors.

A common challenge in the creation of pregnancy cohorts using claims and secondary data is the assignment of the LMP (Thurin et al., 2022; Huybrechts et al., 2019). This information is needed to appropriately define the duration of pregnancy and is not recorded in the majority of these data sources. To address this, we used a previously described algorithm and developed a code definition based on the previous studies (MacDonald et al., 2019; Hornbrook et al., 2007; Margulis et al., 2013). Due to the inability to perform a validation study to assess the accuracy of our estimates, we compared AM-PREGNANT gestational ages with CDC WONDER national estimates. We found comparable results in addition to trends indicating the impact of adopting ICD-10 codes for defining gestational age after 2015. The slight variation in the average gestational age between AM-PREGNANT and CDC WONDER of less than a week was consistent with the observation by Ailes et al. (2023). The results presented in this study suggest that AM-PREGNANT closely mirrors the source population, strengthening the suitability of the cohort in the assessment of exposures and outcomes in perinatal pharmacoepidemiological research. Nevertheless, it will be important, depending on the research question, to conduct sensitivity analyses and acknowledge differences between our study sample (AM-PREGNANT) and the source population to account for potential sources of bias and how we might adjust the estimate for better inferences (Lesko et al., 2022). Our estimates of the average maternal data were also comparable to the US national estimates when using the CDC WONDER database (Centers for Disease Control and Prevention (CDC), 2023c). We acknowledge that the use of external data is not ideal, and discrepancies between national estimates and cohort study findings can arise due to various factors, including study design, population differences, and methodological variations. Moreover, healthcare databases may cover specific populations. Specifically, the percentage of pregnancies covered by MarketScan® may vary depending on the specific cohort or time-period under consideration (Packnet, 2023). Nevertheless, previous research showed that private insurance was the most common principal source of payment for deliveries in 2021 (51.7%), providing coverage for more than half of mothers giving birth in the US (Valenzuela and Osterman, 2023). Therefore, despite the need to cautiously interpret results and consider the broader context of the research question, similar distributions of selected variables reassure the feasibility of the algorithm used for building AM-PREGNANT.

For describing AM-PREGNANT, harmonized definitions aligned with CAMCCO were used, considering its potential inclusion in multi-country studies (Bérard and Sheehy, 2014; Bérard et al., 2022). Recommendations and methods for defining covariates are continuously evolving (Ehrenstein et al., 2024), and the methods adopted in this study reflect the best available knowledge to date. Several other characteristics can be defined using MarketScan®, and it is known that variability in health event occurrences when the same algorithms are applied across different data sources may occur (Ehrenstein et al., 2024). Therefore, the algorithms to operationalize variable definitions might change depending on the research question under study. Definitions used in this project are listed in Supplementary Material. When used in multi-country studies, methods for addressing the differences in distribution of key covariates will be adopted (Webster-Clark et al., 2023; Webster-Clark et al., 2024a; Shu et al., 2023; Webster-Clark et al., 2024b).

Overall, the prevalence of covariates we presented was similar to that reported in previous validated linkage studies (MacDonald et al., 2019; Moll et al., 2021; Sumner et al., 2021; Ailes et al., 2023; Matcho et al., 2018; Hornbrook et al., 2007; Andrade et al., 2017). In terms of prescription medication filled, we found that 77% of pregnancies filled prescriptions for at least one medication, which was within the expected results given that non-prescription medications are unlikely to be captured in an insured population (Centers for Disease Control and Prevention (CDC), 2023b; Mitchell et al., 2011). Studies exploring medication use during pregnancy usually exclude vitamins, vaccines, and other medications that could be acquired over the counter (Mitchell et al., 2011). We chose not to exclude these classes of medications since the aim of our study was to provide a comprehensive overview of medication dispensing in this cohort, for both pregnancies and linked children. The observed increase in prescription medication filled during pregnancy is probably related to the fact that vitamins and pregnancy-related medications were frequently prescribed early in pregnancy, potentially at a higher rate than the rate at which other medications were discontinued. We investigated the trend by excluding vitamins and their combinations, but an increase in use was still observed during pregnancy (Supplementary File S3; Table 2). Previous work had also excluded medications used exclusively topically, locally, or intravenously (Mansour et al., 2024), which could have altered the observed trend. The first trimester is a critical phase in terms of exposure to medication due to the organogenesis period. The patterns of medication dispensing in pregnancy observed in AM-PREGNANT provide the possibility of investigating monotherapy, polytherapy, and other risk factors that may be associated with various perinatal outcomes in both mothers and their children. It should be noted that medication dispensing was observed during the second and third trimesters for spontaneous and planned abortions, which were 13% and 19%, respectively. According to the CDC, the vast majority of abortions occur during the first trimester of a pregnancy (Diamant et al., 2024; Kortsmit et al., 2023). Specifically, in 2021, 93% of abortions occurred during the first trimester (at or before 13 weeks of gestation). An additional 6% occurred between 14 and 20 weeks of pregnancy, and approximately 1% were performed at 21 weeks or more of gestation (Diamant et al., 2024; Kortsmit et al., 2023). Therefore, prescription medication filling observed in the third trimester for the non-live birth outcomes represents prescriptions that overlap with the beginning of the time-window and may reflect the capture of abortions occurring in later gestational ages.

In addition to the prevalence of medication dispensing, we demonstrated the top 10 most frequently filled classes of medications during pregnancy. Anti-infectives were the most prevalent class filled during pregnancy, and this pattern is similar to that of previous pregnancy cohort studies in both Canada and US (Bérard and Sheehy, 2014; Bérard et al., 2022; Mansour et al., 2024). The most prevalent class of prescription medication filled was also anti-infectives for linked children. AM-PREGNANT, therefore, represents an opportunity to assess several outcomes in both mothers and children using these medications.

The overall prevalence of preterm birth in the linked-child cohort was slightly higher than the US national estimates (11.9% vs. 10.1%, 2018–2020) (Centers for Disease Control and Prevention (CDC), Division of Reproductive Health, 2022). This overestimation is expected, as the estimate refers to children who were linked to a pregnancy with available child information. When assessing preterm birth prevalence using a definition in which the denominator included all preterm births among pregnancies ending in a live birth, regardless of linkage to a child, a prevalence of 10.2% was found, which is similar to the US national estimates. We compared this estimate with other Canadian mother–child cohorts, and a higher prevalence was found compared to Canadian estimates (Supplementary File S3; Table 3). A greater proportion of extremely and very preterm birth was observed among children without continuous enrollment. In the US, there is an issue related to the healthcare system in which multiple insurers compete for employment-based insurance contracts and individual enrollees. As a result, turnover among commercial insurance enrollees reflects not only individuals switching insurers but also changes in employment and employers switching insurance providers (MacDonald et al., 2019; Fang et al., 2022). Consequently, dependent enrollees—such as children—are often missed and not properly followed over time, leading to discrepancies when comparing enrolled and non-enrolled populations. The differences observed between AM-PREGNANT and Canadian mother–child cohorts are, therefore, expected, as we are comparing an employed and insured population in the US with a publicly covered population in Canada. The weighting of the cost-benefit to relaxing continuous enrollment among linked children will need to be evaluated for future studies. Other characteristics do not suggest that a sicker population is being excluded, but assumptions would be needed in case these characteristics become outcomes of interest. When using claims US data, mortality data are not available; therefore, it is not known whether these children died or not, and perhaps death was one of the reason for not being continuously enrolled.

For LBW, estimates observed in AM-PREGNANT were half of the US national estimates, 4.7% vs. 8.2% in 2020 (Osterman et al., 2023). It is known that weight at birth is not available in claims databases, including MarketScan® data, where estimates rely exclusively on recorded diagnosis codes. Performance of these codes has been validated by Chomistek et al. (2023) in the Optum Research Database with PPVs close to 100%. Kasman et al. assessed LBW using ICD-9, ICD-10, and DRG codes limited to extreme immaturity and extremely low birth weight. In their study, a 4.7% prevalence of LBW was estimated (2007–2016) (Kasman et al., 2020). We adopted the Agency for Healthcare Improvement (AHRQ) definition, which comprises a broader range of ICD-9 and ICD-10 codes used for assessing pediatric quality indicators in the US healthcare system (Agency for Healthcare Research and Quality (AHRQ), 2023b), and searched for codes in both mothers’ and children’s files. We still have a possible underestimation of this measure. These results reinforce the need for improving algorithms related to infant growth when using administrative databases, as this measure is poorly captured.

Other linked-children characteristics were assessed in AM-PREGNANT, including medication dispensing. Follow-up time of children when using insurance data is limited by continuous enrollment (MacDonald et al., 2019). AM-PREGNANT average follow-up time for continuously enrolled children was 3.3 years, which is a great opportunity for studying acute conditions. When studying long-term outcomes, some limitations may arise.

When evaluating the prevalence of major congenital malformations adopting the harmonized definition used in previous Canadian studies (definition A), higher estimates were observed (13.1% of live births, Table 2) than the US estimates (approximately 4% of live births) (Centers for Disease Control and Prevention CDC, 2008). The estimate decreased to 4% when requiring at least two diagnosis codes recorded on two different days. Huybrechts et al. demonstrated that when major congenital malformations are defined based on a single diagnostic code, the prevalence of malformations exceeds 10%, approximately three times higher than that reported in the US population (approximately 4%) (Huybrechts et al., 2019; Centers for Disease Control and Prevention (CDC), 2008), which is the same as the results we obtained. Nevertheless, we decided to demonstrate the one-code definition in AM-PREGNANT, given that we aimed to use harmonized definitions with QPC and CAMCCO initiative (Bérard and Sheehy, 2014; Bérard et al., 2022). Nevertheless, in future studies using AM-PREGNANT, mainly when adopting a common data model in multi-country studies where major congenital malformations are the outcomes of interest, at least two diagnosis codes, or one diagnosis and related procedure, as previously described (Huybrechts et al., 2019), should be adopted when using the US cohort.

AM-PREGNANT has several strengths. We built upon previous algorithms and assembled the largest and most up-to-date mother–child cohort from a US representative sample of patients with employer-provided health insurance. Inclusion criteria were as follows: 1) continuous enrollment in the insurance plan for a given baseline period; 2) full coverage, including prescription benefits; 3) an appropriate enrollment type (e.g., fee-for-service or capitated plans, provided that they do not underreport encounter claims); and 3) linkage to infants for pregnancies ending in a live birth (Huybrechts et al., 2019). We, therefore, obtained baseline characteristics, including pregnancy outcomes, similar to the US national estimates, demonstrating the feasibility and reliability of this cohort.

Limitations of AM-PREGNANT are similar to those previously reported when constructing pregnancy-linked cohorts using claims data sources. LMP estimation continues to be one of the main concerns when using claims data. The timing of medication exposure during pregnancy is critical when evaluating adverse effects on infant development. Thus, erroneous estimates of LMP will misclassify exposure time. To reduce the risk of inaccurately estimating the LMP, we used previously established and validated codes (Hornbrook et al., 2007; Margulis et al., 2013; Margulis et al., 2023), and as a result, we obtained gestational age estimates similar to the national estimates. The improved specificity of ICD-10 codes enhanced gestational age ascertainment in the later years of the study; however, their limited availability earlier in the study period may have led to incomplete or inconsistent gestational age data. To assess the impact of this transition, we reviewed the distribution of outcomes by gestational age across both coding eras. In AM-PREGNANT, 81% of deliveries (live births, stillbirths, and mixed births) were captured using ICD-10 codes post-2015, compared to 76% using ICD-9 codes before the transition. The prevalence of spontaneous abortions remained consistent at approximately 17% in both periods, suggesting stable classification for this outcome. However, we observed a lower prevalence of elective abortions post-2015 (3%) compared to that in the pre-2015 period (7%), a trend also reported by Sa et al. (2020). This discrepancy may reflect underreporting in claims data, particularly given that a significant proportion of individuals seeking abortion care pay out-of-pocket due to the lack of insurance coverage or privacy concerns. These findings underscore the importance of considering coding system transitions when interpreting trends over time.

Another challenge is related to the identification of pregnancy outcomes other than live births. The adoption of a hierarchical algorithm, similar to those used by MacDonald et al. (2019), Ailes et al. (2016), and Ailes et al. (2023), ensured the identification of pregnancy outcomes similar to the US population for deliveries (i.e., live births, stillbirths, and mixed births) and spontaneous abortions (Benson et al., 2023). The identification of induced/planned abortions appeared to be underestimated; however, this has been an issue even when assessing surveillance data (Kortsmit et al., 2022). Administrative claims data offer various advantages for pharmacoepidemiological research, but bias due to the misclassification of exposure, outcome, and covariates is usually a concern. In terms of exposure to medication, despite acknowledging the difference between filling a prescription for a medication and taking the medication, especially given potential discontinuation once pregnancy is recognized, it is well-known that a strength of using administrative claims to evaluate medication effects is the comprehensive data on prescription fills, which are more reliable than self-reported use or physician-ordered prescriptions. In addition, lack of capture for over-the-counter medications may affect both maternal and child exposures, leading to misclassification in classes of medications used for treating common conditions of pregnancy, such as gastrointestinal diseases (e.g., antacids), fever and pain (e.g., ibuprofen, acetaminophen, and other anti-inflammatory medications), cold, flu, allergies (e.g., anti-histamines and decongestants), and other medications that can be purchased out-of-pocket by the user without the need for a prescription or those that are not covered by insurance (Funk and Landi, 2014). Another limitation is the lack of information on medications dispensed during hospital stays. Although pharmacy claims data may not always accurately reflect actual medication exposure, methods to address such misclassification have been described (Funk and Landi, 2014; Walraven, 2018) and are increasingly used (Brown et al., 2024; Petersen et al., 2021). However, because MarketScan® data do not capture inpatient medication dispensing, there is potential for exposure misclassification and confounding by indication, which cannot be fully addressed in the analysis. As such, research questions that rely specifically on in-hospital medication data should be avoided. Regarding generalizability, the MarketScan® population likely includes healthier pregnant individuals with private insurance coverage, which may lead to more favorable outcomes. Therefore, findings are generalizable to the privately insured population but may not be applicable to individuals covered by Medicaid or other public insurance programs. Johnson et al. (2023) demonstrated that 42.1% of all births in the US in 2019 were covered by Medicaid, indicating that nearly half of the population may experience higher rates of inadequate or delayed prenatal care—factors known to be associated with adverse infant outcomes. This is a methodological concern that will be taken into account when conducting multi-country studies using AM-PREGNANT. Although this cohort is representative of the US pregnant population, these differences in terms of disparities are an opportunity to triangulate results using data from different settings. The practice of strengthening causal inferences by integrating results from multiple approaches –each with different and largely unrelated sources of potential bias, was employed by Lawlor et al. (2016), including the use of different sources. Methods for pooling results considering the external validity when using distributed data networks were described already (Webster-Clark et al., 2023), and these approaches will be taken into account for future studies.

## 5 Future directions

AM-PREGNANT represents a reliable and promising resource for addressing queries and research questions on the safety and effectiveness related to medication use during pregnancy. By aggregating harmonized data from multiple populations, namely, the US and Canada (with CAMCCO DATA), we can increase statistical power to detect associations, which would, in turn, allow us to answer novel research questions as they pertain to rare exposures and outcomes, as is generally the case in perinatal pharmacoepidemiology. By following successful initiatives such as the ConcePTION Common Data Model (Thurin et al., 2022), in addition to applying appropriate methods when using different data sources from different countries and contexts, we can triangulate results to strengthen the evidence for answering causal questions (Lawlor et al., 2016). AM-PREGNANT will provide data on privately insured subjects, allowing comparisons between Canada and the US, as well as across Canadian provinces—an aspect rarely explored in other perinatal epidemiology studies. Our cohort will add value to the research program by identifying and quantifying differences in prescribing practices and medication dispensing during pregnancy and childhood across Canada, its provinces, and the US, and it will help in identifying the risk profiles of prescription medications filled during pregnancy and in the pediatric populations.

It is important to note that the prevalence of chronic conditions such as depression and hyperglycemia varies significantly across regions and populations globally. As such, trends in prescription medication use may reflect not only true changes in disease burden but also differences in healthcare systems, diagnostic practices, and access to care. Future studies should explore regional and international variations to better understand the broader applicability of our findings in other contexts, such as the evaluation of medications and their outcomes in pregnancy and children living in developing countries.

## 6 Conclusion

We have assembled AM-PREGNANT, which represents an important resource for the assessment of prescription medication safety for both mothers and their children. The large numbers of individuals included for both mothers and the linked mother–child cohort, even when using a conservative continuous enrollment requirement, provide an excellent resource for assessing rare exposures and outcomes. Preterm birth rates, mean maternal age, and gestational ages were comparable to US population estimates, reassuring the validity of this cohort in terms of feasibility, reliability, and generalizability. AM-PREGNANT represents an additional data source to be incorporated when performing multi-country studies in the field of perinatal pharmacoepidemiology.

## Data Availability

The datasets presented in this article are not readily available due to confidentiality agreements. Requests to access MarketScan data can be made through: https://www.merative.com/real-world-evidence/real-world-data-analytics.
