# Supplementary material for: The AMerican PREGNANcy Mother–Child CohorT: description and prevalence of baseline outcomes and medication dispensing
Source: Front Pharmacol. 2025 Aug 5;16:1608403. doi: 10.3389/fphar.2025.1608403 (PMC12361823; doi:10.3389/fphar.2025.1608403)
Supplement: Supplementary file 2 [file Supplementaryfile3.docx]

Supplementary File S3

# Supplementary Table 1. Maternal characteristics of AM-PREGNANT (from Merative™ MarketScan® Commercial Database, USA) by linked and non-linked pregnancies,* 2004-2020.

| **Characteristics** | **All deliveries 2004-2020^*,**^** | **Mother-child linked cohort** | **Non-linked  cohort** |
| --- | --- | --- | --- |
|  | (n=3,663,521) | (n=2,554,964 ) | (n=1,108,557) |
| **Calendar year at end of pregnancy** |  |  |  |
| 2004 | 100,323 (2.7%) | 75,677 ( 3.0 %) | 24,646 ( 2.2%) |
| 2005 | 134,174 (3.7%) | 101,651 ( 4.0 %) | 32,523 ( 2.9%) |
| 2006 | 126,022 (3.4%) | 96,503 ( 3.8 %) | 29,519 ( 2.7%) |
| 2007 | 200,951 (5.5%) | 149,907 ( 5.9 %) | 51,044 ( 4.6%) |
| 2008 | 217,605 (5.9%) | 154,849 ( 6.1 %) | 62,756 ( 5.7%) |
| 2009 | 294,716 (8.0%) | 202,325 ( 7.9 %) | 92,391 (8.3%) |
| 2010 | 287,795 (7.9%) | 196,867 ( 7.7 %) | 90,928 (8.2%) |
| 2011 | 315,108 (8.6%) | 219,876 ( 8.6 %) | 95,232 (8.6%) |
| 2012 | 348,905 (9.5%) | 238,569 ( 9.3 %) | 110,336 (10.0%) |
| 2013 | 281,845 (7.7%) | 188,662 ( 7.4 %) | 93,183 (8.4%) |
| 2014 | 282,922 (7.7%) | 191,081 ( 7.5 %) | 91,841 (8.3%) |
| 2015 | 206,453 (5.6%) | 137,599 ( 5.4 %) | 68,854 (6.2%) |
| 2016 | 224,290 (6.1%) | 152,423 ( 6.0 %) | 71,867 (6.5%) |
| 2017 | 196,255 (5.4%) | 136,208 ( 5.3 %) | 60,047 (5.4%) |
| 2018 | 179,209 (4.9%) | 127,281 ( 5.0 %) | 51,928 (4.7%) |
| 2019 | 142,251 (3.9%) | 98,290 ( 3.8 %) | 43,961 (4.0%) |
| 2020 | 124,697 (3.4%) | 87,196 ( 3.4 %) | 37,501 (3.4%) |
| **Region** |  |  |  |
| Northeast | 563,995 (15.4%) | 414,436 ( 16.2 %) | 149,559 ( 13.5 %) |
| North Central | 832,150 (22.7%) | 610,697 ( 23.9 %) | 221,453 ( 20.0 %) |
| South | 1,518,741 (41.5%) | 1,022,258 ( 40.0 %) | 496,483 ( 44.8 %) |
| West | 697,387 (19.0%) | 476,478 ( 18.6 %) | 220,909 ( 19.9 %) |
| Unknown | 51,248 (1.4%) | 31,095 ( 1.2 %) | 20,153 ( 1.8 %) |
| **Maternal Age at the end of pregnancy, years** | |  |  |
| Mean (SD) | 30.4 (5.4) | 31.5 (4.6) | 28.0 (6.2) |
| <20 | 109,525 (3.0%) | 9,827 ( 0.4 %) | 99,698 ( 9.0 %) |
| 20-34 | 2,723,680 (74.4%) | 1,895,998 ( 74.2 %) | 827,682 ( 74.7 %) |
| 35-40 | 739,084 (20.2%) | 579,829 ( 22.7 %) | 159,255 ( 14.4 %) |
| >40 | 91,232 (2.5%) | 69,310 ( 2.7 %) | 21,922 ( 2.0 %) |
| **Estimated Gestational Age, weeks** |  |  |  |
| Mean (SD) | 38.6 (2.0) | 38.6 (2.0) | 38.6 (1.9) |
| **Multi-fetal pregnancies** | 96,664 (2.6%) | 72,110 ( 2.8 %) | 24,554 (2.2 %) |
| **Previous Caesarean delivery** | 597,523 (16.3%) | 129,085 ( 5.0 %) | 41,472 (3.7 %) |
| **Alcohol/substance abuse** | 54,757 (1.5%) | 28,297 ( 1.1 %) | 26,460 (2.4 %) |
| **Tobacco use** | 37,678 (1.0%) | 16,286 (0.6%) | 21,536 (1.9%) |
| **Maternal comorbidities** |  |  |  |
| Thyroid disorders | 332,924 (9.1%) | 251,992 ( 9.9 %) | 80,932 (7.3 %) |
| Depression | 203,869 (5.6%) | 269,585 ( 10.5 %) | 130,912 (11.8 %) |
| Hypertension | 206,456 (5.6%) | 303,464 (11.7 %) | 122,445 (11.5 %) |
| Asthma | 185,505 (5.1%) | 222,007 ( 8.6 %) | 93,495 (8.4 %) |
| Diabetes | 135,013 (3.7%) | 437,843 ( 17.0 %) | 141,992 (12.8 %) |
| Epilepsy | 11,454 (0.3%) | 7,145 ( 0.3 %) | 4,309 (0.4 %) |
| Autoimune diseases | 6,8260 (1.9%) | 49,484 (1.9%) | 19,282 (1.7%) |
| Infections | 431,539 (11.8%) | 278040 (10.8%) | 156,046 (14.1%) |
| Obesity | 219,816 (6.0%) | 146,807 (5.7%) | 74,787 (6.8%) |
| Renal diseases | 30,067 (0.8%) | 20,800 (0.8%) | 9,537 (0.9%) |
| **Medication use overall***** | 2,900,679 (79.2%) | 2,038,838(79.80%) | 861,841 (77.7%) |
| Medication use - 90 days before pregnancy | 1,834,093 (63.2%) | 1,304,529 (64.0%) | 529,564 (47.8%) |
| Medication use - during pregnancy | 2,573,443 (88.7%) | 1,802,360 (88.4%) | 771,083 (69.6%) |
| First trimester | 2,113,450 (82.1%) | 1,499,756 (83.2%) | 613,694 (79.6%) |
| Second trimester | 1,876,847 (72.9%) | 1,321,767 (73.3%) | 555,080 (72.0%) |
| Third trimester | 1,919,418 (74.6%) | 1,351,658 (75.0%) | 567,760 (73.6%) |
| Medication use - 42 days after pregnancy | 2,405,181 (82.9%) | 1,719,481 (84.3%) | 685,700 (61.9%) |

Legend: SD= Standard Deviation

* From deliveries, that included livebirth, stillbirth and mixed births

** May represent more than one pregnancy per person

*** Measured by claimed filled prescriptions from 90 days before pregnancy until postnatal period

**Supplementary Figure 1. Average maternal age compared to CDC WONDER estimates.**


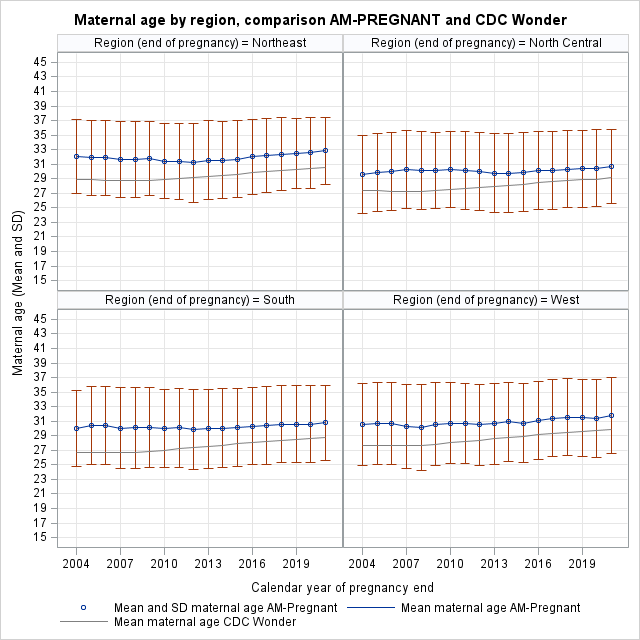


**Supplementary Figure 2. Average gestational age compared to CDC WONDER estimates.**


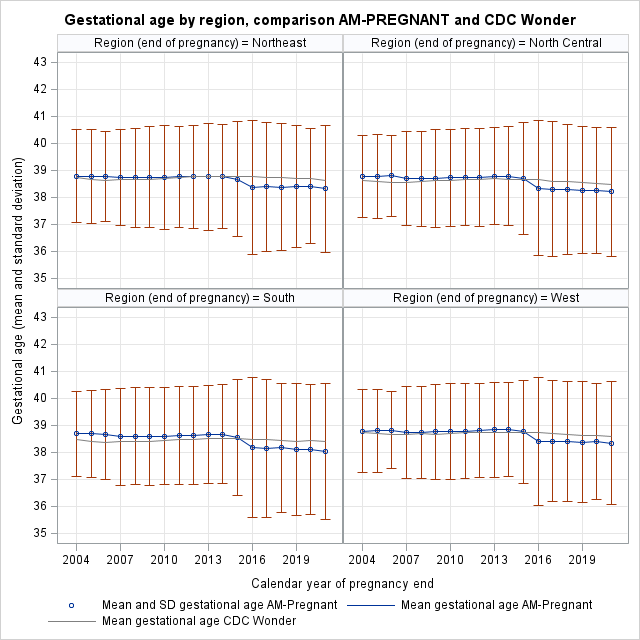


**Supplementary Table 2. Prevalence of medication dispensing comparing deliveries dispensed vitamins and combinations with deliveries without vitamins and combinations dispensing.**

| **Medication dispensing** | **Deliveries including vitamins and combinations** | | **Deliveries without vitamins and combinations** | |
| --- | --- | --- | --- | --- |
|  | (n=3,663,521) | (%) | (n=3,663,521) | (%) |
| Overall | 2,900,679 | 79.2 | 2,850,010 | 77.8 |
| 90 days before pregnancy | 1,834,093 | 63.2 | 1,768,904 | 62.1 |
| During pregnancy | 2,573,443 | 88.7 | 2,396,039 | 84.1 |
| First trimester | 2,113,450 | 82.1 | 1,863,189 | 77.8 |
| Second trimester | 1,876,847 | 72.9 | 1,480,196 | 61.8 |
| Third trimester | 1,919,418 | 74.6 | 1,569,137 | 65.5 |
| 42 days after pregnancy | 2,405,181 | 82.9 | 2,282,833 | 80.1 |

# Supplementary Table 3. Canadians (CAMCCO: Quebec, Manitoba, Saskatchewan, and Alberta) and US (AM-PREGNANT) mother-child cohorts – Overall prevalence of prematurity, LBW, multiplicity, and medication use during pregnancy.

|  | Alberta | Manitoba | Saskatchewan | Québec | United States |
| --- | --- | --- | --- | --- | --- |
| Characteristcs | 2005-2018 | 1995-2018 | 1996-2020 | 1998-2015 | 2004-2020 |
|  | (n=688,270) | (n=347,888) | (n=291,324) | (n=248,787) | (n=4,767,208) |
| Prematurity (gestational age <37 weeks gestation)* | 6.8% | 6.5% | 5.9% | 6.6% | 10.2% |
| Extremely preterm (< 28 weeks) | 6.7% | 6.0% | 6.6% | 4.6% | 2.6% |
| Very preterm (28-<32 weeks) | 7.8% | 8.1% | 8.0% | 7.6% | 6.4% |
| Moderate to late preterm (32-<37 weeks) | 85.5% | 85.9% | 85.4% | 87.8% | 91.0% |
| Low birth weigth | 5.2% | 4.3% | 4.0% | 5.1% | 3.8% |
| Multiplicity | 1.9% | 2.5% | 1.3% | 1.0% | 2.6% |
| Major congenital malformations** | 7-8% | 5-3% | 5-11% | 7-11% | 13.1% |
| Medication use during pregnancy*** | - | - | - | 59.0% | 86.7% |

* Measured among pregnancies ending in live births (n=3,647,001), according to WHO defintion.

** Numbers are prevalence ranges, overall prevalences were not available in CAMCCO.

*** The overall prevalence of medication use during pregnancy in CAMCCO corresponds to 61% (1) , individual estimates for Alberta, Manitoba, and Saskatchewan were not available.

1. Bérard A, Kaul P, Eltonsy S, Winquist B, Chateau D, Hawken S, et al. The Canadian Mother-Child Cohort Active Surveillance Initiative (CAMCCO): Comparisons between Quebec, Manitoba, Saskatchewan, and Alberta. PLoS One. 2022;17(9):e0274355.
